# Genetic variants associated with alcohol dependence co-ordinate regulation of *ADH* genes in gastrointestinal and adipose tissues

**DOI:** 10.1038/s41598-020-66048-z

**Published:** 2020-06-18

**Authors:** Rebecca Hibberd, Evgeniia Golovina, Sophie Farrow, Justin M. O’Sullivan

**Affiliations:** 1 Liggins Institute, The University of Auckland, Auckland, New Zealand; 2 MRC Lifecourse Epidemiology Unit, University of Southampton, Southampton, United Kingdom; 3 A Better Start National Science Challenge, Auckland, New Zealand; 4 Natural Sciences, Faculty of Environmental and Life Sciences, University of Southampton, Southampton, United Kingdom

**Keywords:** Behavioural genetics, Gene expression, Gene regulation, Genetic association study, Genetic markers, Addiction

## Abstract

GWAS studies have identified genetic variants associated with Alcohol Dependence (AD), but how they link to genes, their regulation and disease traits, remains largely unexplored. Here we integrated information on the 3D genome organization with expression quantitative loci (eQTLs) analysis, using CoDeS3D, to identify the functional impacts of single nucleotide polymorphisms associated with AD (p < 1 × 10^−6^). We report that 42% of the 285 significant tissue-specific regulatory interactions we identify were associated with four genes encoding Alcohol Dehydrogenase - *ADH1A*, *ADH1B*, *ADH1C* and *ADH4*. Identified eQTLs produced a co-ordinated regulatory action between *ADH* genes, especially between *ADH1A* and *ADH1C* within the subcutaneous adipose and gastrointestinal tissues. Five eQTLs were associated with regulatory motif alterations and tissue-specific histone marks consistent with these variants falling in enhancer and promoter regions. By contrast, few regulatory connections were identified in the stomach and liver. This suggests that changes in gene regulation associated with AD are linked to changes in tissues other than the primary sites of alcohol absorption and metabolism. Future work to functionally characterise the putative regulatory regions we have identified and their links to metabolic and regulatory changes in genes will improve our mechanistic understanding of AD disease development and progression.

## Introduction

Alcohol dependence (AD) is distinct from the responsible social use of alcohol. Rather, AD is a medical condition that is characterised by “craving, tolerance, a preoccupation with alcohol and continued drinking, in spite of harmful consequences” (NICE)^1^. AD contributes to the 5.1% of world-wide disease burden with which alcohol is associated (WHO)^2^. Common medical conditions associated with AD include physical conditions such as heart disease, hypertension and a range of gastrointestinal cancers^3^, as well as mental and psychiatric disorders such as major depressive disorder, schizophrenia and bipolar disorder^4^. In addition to its medical implications, AD has been linked to increases in antisocial behaviour in young adults^5^ and crime incidence^6^, adding to the economic costs of the condition.

AD is a clinical diagnosis and the severity of the condition presents as a continuum across populations. Both genetic and environmental factors impact an individual’s likelihood of developing the condition, with twin-studies estimating the heritability of alcohol use disorders to be around 50%^7^. Genetic variants that are associated with, and therefore likely predispose to, AD have been identified^8–13^. However, the impacts of these genetic variants on metabolism and alcohol detoxification, and the mechanisms driving such impacts, remain poorly understood.

Ethanol, the alcohol present in ‘alcoholic’ beverages, is a toxic substance which is primarily removed from the body via its oxidation in the liver. Ethanol is absorbed through the stomach and small intestine into the blood which is circulated to the liver via the hepatic portal vein for filtering^14^. The liver is generally recognised as the main site for alcohol metabolism^15^. First pass metabolism also occurs in the gastric system and depends on the activity of enzymes and the rate of gastric emptying^14^. After first pass metabolism, ethanol is distributed around the body by the blood to various tissues where it can also be metabolised in the cytosol of cells^14^.

The main pathway of ethanol breakdown involves the oxidation of ethanol to acetaldehyde, mediated by alcohol dehydrogenase (ADH). Due to the toxicity of acetaldehyde at high or sustained concentrations^16^, a second reaction oxidises acetaldehyde to acetate, catalysed by aldehyde dehydrogenase (ALDH). There are seven genes coding for ADH isozymes, aligned head to tail on chromosome 4. ADH isozymes are very highly transcribed in the liver (excluding ADH7; GTEx version 8^17^). Other enzymes have minor roles in alcohol metabolism, for example CYP2E1 is part of the liver cytochrome P450 enzyme system, and catalase^14,18,19^.

Although the genes coding ADH and ALDH are known to be important in alcohol metabolism^10,14,20,21^, the way in which their expression is regulated in different tissues is not fully understood. Studies have identified alleles in *ADH* and *ALDH* that have protective effects against developing AD^22–24^. Similarly, comparative population studies have highlighted the roles of different alleles in AD disease risk (reviewed in Dasgupta 2015^14^).

Genome wide association studies (GWAS) search the entire human genome to identify genetic markers (i.e. Single Nucleotide Polymorphisms [SNPs]) that are associated with and may even be causal for a particular phenotype. GWAS incorporate no prior knowledge of the SNPs, meaning that there is no assigned functional information. Thus, all variants have an equal likelihood of being causally associated with the tested phenotype to start with. Despite GWAS studies into AD identifying SNPs that confirm a role for genetic variation^8–13^, the individual and co-operative mechanism through which these variants impact an individual’s disease risk remain largely unknown. The functional characterisation of these variants is complicated by the fact that most of the SNPs are present in non-coding (intergenic and intronic) regions of the genome^25–27^. It is likely that many of these variants affect the regulation of metabolic genes consistent with observations for different phenotypes^26,28–30^. Regulatory roles for these alcohol dependent variants could impact a wide range of biological pathways in different ways and to different extents in different tissues. The infinitesimal hypothesis (Fisher)^31^ argues that the impact of each variant is small, requiring numerous changes to affect a phenotype. Incorporating expression quantitative trait loci (eQTL) and chromatin structural data (i.e. Hi-C) into SNP analyses can help to inform on tissue specific regulatory impacts for SNPs^28,32–34^. Recent studies have taken advantage of these novel methods in a range of pathophysiological contexts to determine the collective impacts of the genetic variation on tissue specific gene regulation^33,35–38^. These novel approaches hold the key to understanding the role that genetic variation plays in AD, potential therapeutic and preventative treatments.

Here, we interrogate the long-range regulatory effects of 73 GWAS SNPs associated with AD^39^. By integrating data on the functions of known genes, tissue specific expression, regulatory elements, and structural genome data, we identify how SNPs may individually and collaboratively impact the pathophysiology of AD.

## Results

### AD-associated GWAS SNPs mark spatial regulatory eQTLs

SNPs (n = 73) associated with AD (*p* < 1 × 10^−6^) were obtained from the NHGRI-EBI GWAS Catalog^39^ (www.ebi.ac.uk/gwas/; Supplementary Table 1). Of the 73 SNPs, 6 were coding variants (4 missense, 2 synonymous) and 67 were non-coding variants (Supplementary Table 1). CoDeS3D^40^ was used to interrogate 3D genome structure data (Supplementary Fig. 1; Supplementary Table 2) to identify tissue-specific spatial eQTLs (and their associated gene – “eGene”; GTEx multi-tissue dataset version 7, Supplementary Table 3) for 34 SNPs. The 34 eQTLs (16 intronic, 9 intergenic, 3 exonic, 3 ncRNA intronic, 1 upstream, 2 UTR3; Supplementary Fig. 2) were involved in 274 cis- and 11 trans-acting regulatory interactions between 72 statistically significant (FDR < 0.05; Benjamini Hotchberg^40^) SNP-eGene pairs across 48 different human tissues (Supplementary Table 4). Nine of the trans-regulated eGenes are loss of function tolerant (pLI = 0.00–0.10, gnomAD v2.1.1^41^), whilst *GREBL1L* is severely loss of function intolerant (pLI = 1, gnomAD v2.1.1^41^). *TPT1-AS1* had no data available. For example, rs2094081 was associated with trans-acting down-regulation of *CYP4B1* (pLI = 0.00, gnomAD v2.1.1^41^) in stomach tissue. *CYP4B1* is a member of the Cytochrome P450 superfamily of enzymes, which contribute to an alternative pathway for alcohol metabolism^14,18^.

24 interactions we identified were associated with regulation of 12 eGenes (including *WDR5B*, *FABP3, KCNJ6* and *ADH1C*) in 9 different brain tissues, of which two were trans-acting (Supplementary Table 4). Notably, the trans-acting interaction with rs7913179 was associated with upregulation of *GFRA2* (pLI = 0.1, gnomAD v2.1.1^41^) in brain amygdala. *GFRA2* encodes glial cell line-derived neurotrophic factors (GDNF) that play a role in different biological processes including neurons cell survival and neurite outgrowth^42^. Signaling by GDNF promotes the survival of dopaminergic neurons^42^ and has been considered as a therapeutic target for the treatment of alcoholism^43^.

We performed gene ontology (GO) and pathway analyses using g:Profiler (the g:GOSt module)^44^ on the genes affected by the eQTLs. We identified 17 molecular function categories (including “alcohol dehydrogenase activity”), 25 biological process categories (including “ethanol oxidation” and “ethanol metabolic process”) and 27 cellular components were significantly enriched (*p* < 0.05, adjusted using the SCS algorithm^44^; Supplementary Table 5) for eGenes. Pathway analysis identified eight significantly enriched biological pathways (including fatty acid degradation and metabolic pathways) associated with AD (*p* < 0.05, adjusted by the SCS algorithm^44^; Supplementary Table 6). These findings provide further evidence supporting the importance of ethanol oxidation, lipid storage and metabolism in the pathophysiology of alcohol disorders such as AD^18,45^.

Despite the pivotal role of the liver in ethanol metabolism^15,18^, only one eQTL exhibited significant impact on gene expression in the liver (rs4699741-*SCARB2*) (Supplementary Table 4, Supplementary Fig. 3). This was the only eQTL within the *ADH* region that exhibited a significant regulatory effect on a gene other than *ADH*. Notably the presence of the alternate allele upregulated *SCARB2* expression through trans-regulation. *SCARB2* is loss of function tolerant (pLI = 0.00; gnomAD v2.1.1^41^).

**Figure 1:**
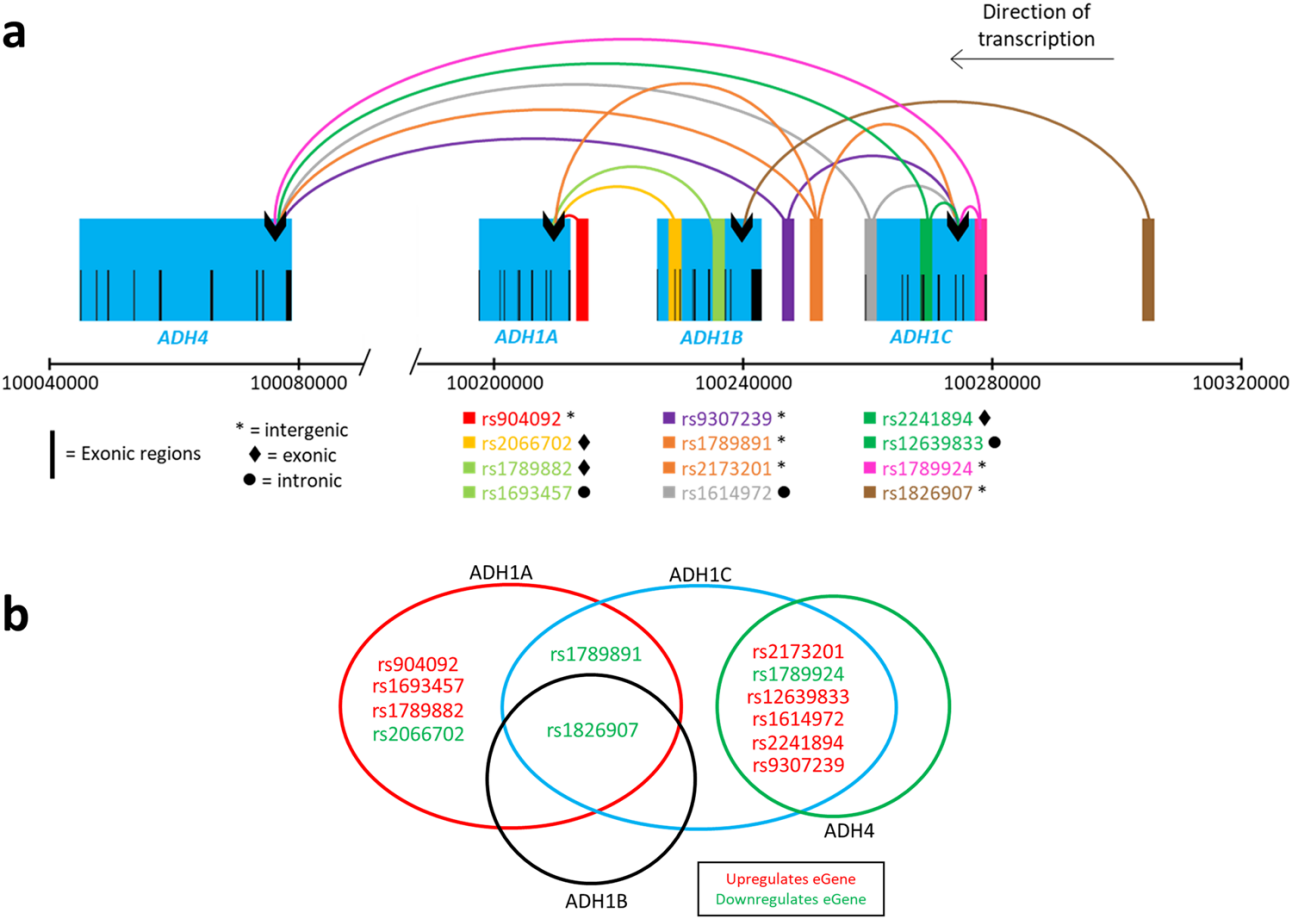
The 12 eQTLs identified as having regulatory interactions with *ADH* genes. Three SNPs located within the *ADH* gene cluster did not interact with *ADH* genes, so are excluded. Of these, one interacted with another gene, *SCARB2*, whilst the other two were not identified as eQTLs. (**a**) Spatial eQTL-eGene interactions within the *ADH* gene cluster (Chromosome 4). ♦●*symbols represent functional annotations obtained from wANNOVAR^59,60^. For simplicity, SNPs separated by <2 kbp in the linear sequence were grouped with the same colour. Genomic locations are according to human genome hg19. The eQTL-eGene interaction analysis used GTEx v7^17^. (**b**) Cross-over of eQTL-eGene interactions in the *ADH* gene region. Red text denotes the SNP is associated with upregulation of the eGene, whereas green text denotes associated downregulation. Multiple variants are associated with co-ordinated upregulation of *ADH1A* or *ADH4* and *ADH1C*.

A 100 kb locus on chromosome 4 (chr4:100210000–100310000), encompassing the *ADH* genes, contained 38% (n = 13) of the eQTLs we identified (Supplementary Fig. 2). 12 of these eQTLs (3 intronic, 4 intergenic, 3 exonic, 1 upstream, 1 ncRNA intronic) and ~42% of regulatory interactions (n = 119) were associated with regulation of the *ADH* genes. In total there were 21 statistically significant eQTL-eGene pairs involving *ADH* genes with a total of 118 cis-acting regulatory interactions across 24 different human tissues (Table 1, Supplementary Fig. 4). These interactions involved four of the seven *ADH* genes within the locus: *ADH1A*, *ADH1B*, *ADH1C* and *ADH4*. Notably, the 3 exonic SNPs that were tested and had significant eQTLs were located within the *ADH* gene region (Supplementary Table 4, Supplementary Fig. 2). Two SNPs (rs1229984, rs17028615) located within the *ADH* locus that were not associated with altered ADH gene expression, also did not exhibit any significant regulatory effects on other genes. eQTLs within the *ADH* locus were associated with a range of tissue-specific regulatory interactions and effect sizes with the four *ADH* genes (Fig. 1a; Table 1). None of the SNPs that were eQTLs for the *ADH* genes exhibited significant eQTLs with any other genes. There were no AD-associated SNPs located within the *ADH1A* or *ADH4* genes (Fig. 1a). rs1826907 was the only eQTL associated with regulation of three *ADH* genes - *ADH1A*, *ADH1B* and *ADH1C* (Fig. 1b). By contrast, 11 SNPs either associated with regulation of *ADH1A*, *ADH1A* and *ADH1C*, or *ADH1C* and *ADH4* simultaneously – but not necessarily in the same tissues (Fig. 1a,b, and Table 1). Four eQTLs (rs1789891, rs1789924, rs1826907 and rs2066702) were associated with downregulation of at least one *ADH* gene in subcutaneous adipose tissue (Fig. 1a, Table 1).

**Table 1:** Significant eQTL-tissue regulatory interactions associated with the *ADH* genes located on Chromosome 4. SNP ID and effect size are shown for each interaction.

Tissue	Gene	SNP ID	Effect Size	SNP ID	Effect Size	SNP ID	Effect Size	SNP ID	Effect Size	SNP ID	Effect Size	SNP ID	Effect Size
Adipose Subcutaneous	*ADH1A*	rs1693457	0.638	rs1789882	0.638	rs1789891	−0.342	rs2066702	−0.696	rs904092	0.634		
	*ADH1B*	rs1826907	−0.174										
	*ADH1C*	rs12639833	0.252	rs1614972	0.255	rs1789891	−0.321	rs1789924	−0.459	rs2173201	0.252	rs2241894	0.256
	*ADH4*	rs1789924	−0.26										
Adipose Visceral Omentum	*ADH1C*	rs1789924	−0.409										
Adrenal Gland	*ADH1A*	rs1693457	0.539	rs1789882	0.54	rs904092	0.54						
Artery Aorta	*ADH1A*	rs1693457	0.691	rs1789882	0.691	rs1789891	−0.484	rs904092	0.692				
	*ADH1B*	rs1826907	−0.296										
Artery Tibial	*ADH1A*	rs1693457	0.479	rs1789882	0.49	rs904092	0.489						
	*ADH1B*	rs1826907	−0.298										
	*ADH1C*	rs1789891	−0.279	rs1789924	−0.26								
Brain Caudate basal ganglia	*ADH1C*	rs12639833	0.632	rs2173201	0.634	rs2241894	0.623						
Breast Mammary Tissue	*ADH1A*	rs1693457	0.491	rs1789882	0.491	rs904092	0.491						
	*ADH1C*	rs1789924	−0.385										
Cells Transformed fibroblasts	*ADH1A*	rs1693457	0.336	rs1789882	0.336	rs1826907	−0.272	rs904092	0.343				
	*ADH1B*	rs1826907	−0.166										
	*ADH1C*	rs1826907	−0.224										
Colon Sigmoid	*ADH1A*	rs1693457	0.636	rs1789882	0.636	rs904092	0.636						
	*ADH1C*	rs1789924	−0.383										
Colon Transverse	*ADH1A*	rs1693457	0.447	rs1789882	0.433								
Esophagus Gastroesophageal Junction	*ADH1A*	rs1693457	0.424										
Esophagus Mucosa	*ADH1C*	rs12639833	0.371	rs1614972	0.284	rs1789891	−0.285	rs1789924	−0.308	rs2173201	0.375	rs2241894	0.373
Esophagus Muscularis	*ADH1A*	rs1693457	0.438	rs1789882	0.438	rs904092	0.427						
	*ADH1C*	rs1789924	−0.273										
	*ADH4*	rs12639833	0.387	rs2173201	0.404	rs2241894	0.393						
Heart Atrial Appendage	*ADH1A*	rs1693457	0.577	rs1789882	0.586	rs904092	0.586						
	*ADH1C*	rs12639833	0.328	rs1789891	−0.501	rs1789924	−0.508	rs2173201	0.332	rs2241894	0.332		
Heart Left Ventricle	*ADH1A*	rs1693457	0.476	rs1789882	0.471	rs904092	0.458						
	*ADH1C*	rs1789924	−0.362										
Lung	*ADH1A*	rs1693457	0.646	rs1789882	0.648	rs904092	0.648						
	*ADH1C*	rs12639833	0.336	rs1614972	0.289	rs1789924	−0.266	rs2241894	0.337	rs9307239	0.216	rs2173201	0.338
Muscle Skeletal	*ADH1B*	rs1826907	−0.24										
	*ADH4*	rs12639833	0.31	rs1614972	0.256	rs2173201	0.305	rs2241894	0.305	rs9307239	0.237		
Nerve Tibial	*ADH1A*	rs1693457	0.372	rs1789882	0.372	rs904092	0.373						
	*ADH1B*	rs1826907	−0.301										
	*ADH1C*	rs1789891	−0.297	rs1789924	−0.221								
Pancreas	*ADH1C*	rs12639833	0.53	rs2173201	0.534	rs2241894	0.536						
Skin Not Sun Exposed Suprapubic	*ADH1A*	rs1693457	0.327	rs1789882	0.342	rs904092	0.344						
	*ADH1C*	rs1789924	−0.253										
Skin Sun Exposed Lower leg	*ADH1A*	rs1693457	0.461	rs1789882	0.463	rs904092	0.457						
	*ADH1C*	rs12639833	0.233	rs1789924	−0.257	rs2173201	0.236	rs2241894	0.239				
Small Intestine Terminal Ileum	*ADH1A*	rs1693457	0.727	rs1789882	0.727	rs904092	0.74						
Spleen	*ADH1C*	rs12639833	0.588	rs2173201	0.572	rs2241894	0.602						
Thyroid	*ADH1A*	rs1693457	0.441	rs1789882	0.44	rs904092	0.444						
	*ADH1B*	rs1826907	−0.217										
	*ADH1C*	rs1789924	−0.264

### *ADH* regulation in adipose tissue

**Figure 2:**
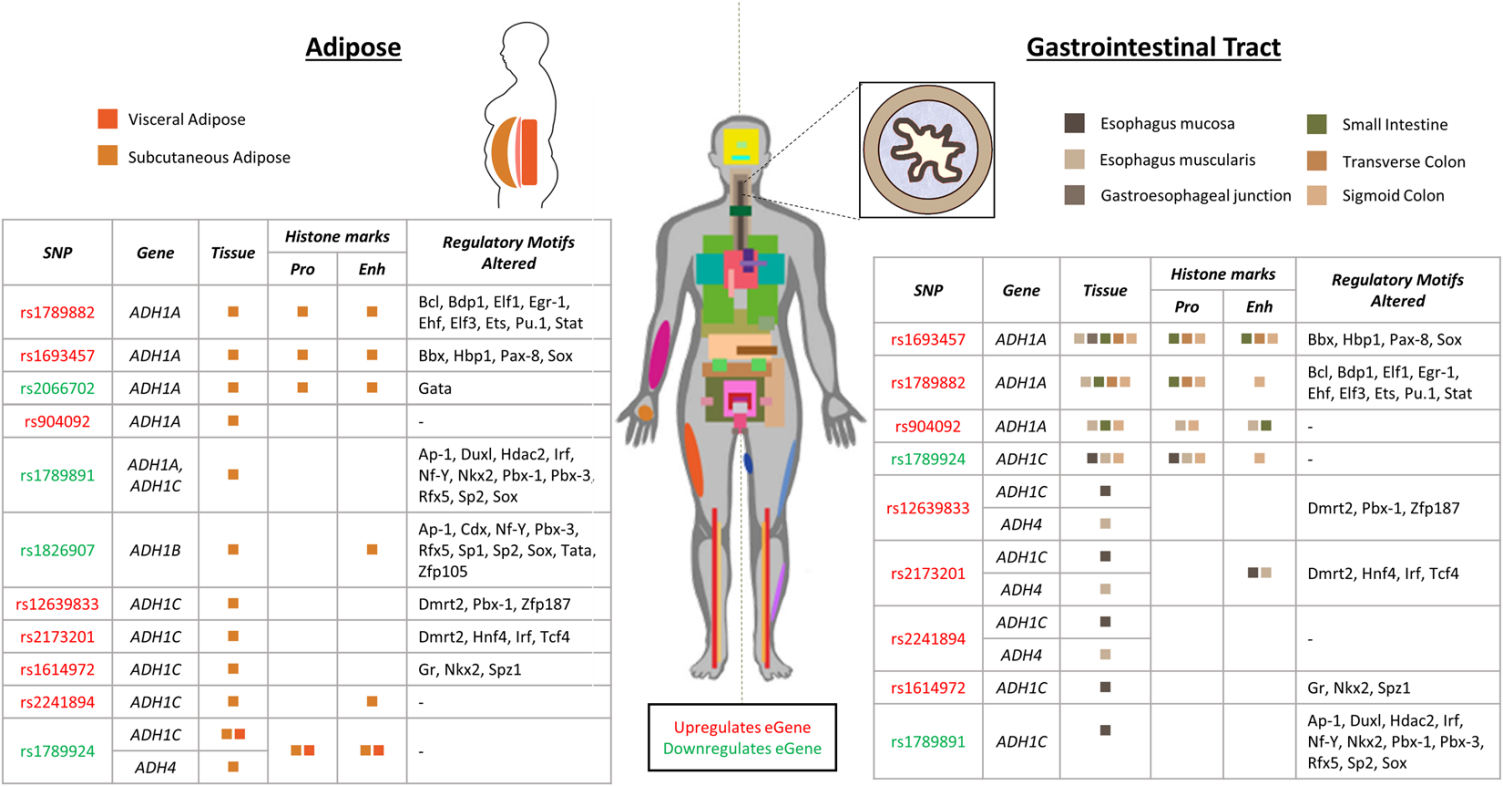
Adipose and gastrointestinal tract tissues are of interest due to their known roles in alcohol metabolism and have many significant eQTL interactions with *ADH* genes. SNPs affect regulatory motifs and are present in areas of the genome that have tissue-specific histone marks that infer some regulatory function. Coloured boxes represent the presence of an eQTL for the listed eGene in the tissue and found tissue-specific histone marks for the SNP. Tissues are represented by the same colour in the table and diagrams. Enh = enhancer histone marks; Pro = promoter histone marks. H3K4ac and H3K27ac are grouped as enhancer histone marks and H3K4me3 and H3K9ac as promoter histone marks. Histone marks and regulatory motif alterations were obtained from HaploReg (version 4.1)^49,50^. Red text denotes the SNP is associated with upregulation of the eGene, whereas green text denotes associated downregulation.

Fatty Liver Disease has direct links to chronic alcohol consumption through the mobilisation of adipose tissue^19,46,47^. Moreover, subcutaneous adipose tissue is thought to impact the secondary metabolism of ethanol after the liver. We observed variant specific impacts on adipose tissue at the *ADH* locus (Fig. 2 and Table 1). Subcutaneous adipose has the most eQTL interactions involving *ADH* genes of any tissue, with 13 regulatory interactions across 11 of the 12 SNPs. It is the only tissue in which we identified eQTLs for all four *ADH* genes (Table 1, Supplementary Fig. 4, Supplementary Table 4). By contrast, only one eQTL, involving *ADH1C* and rs1789924, was identifiable in visceral adipose.

Epigenetic modifications (e.g. H3K4ac, H3K27ac, H3K4me3 and H3K9me3) are recognised as marking promoter and enhancer regions^48^. To further investigate the regulatory activity of the 12 significant eQTLs associated with *ADH* genes in subcutaneous adipose, we obtained data on promoter and enhancer histone marks, DNase marks and regulatory motif alterations from HaploReg (version 4.1)^49,50^ (Fig. 2, Supplementary Table 7). The locus marked by rs1826907 was associated with down regulation of *ADH1B* interaction in subcutaneous adipose (Fig. 2). HaploReg^49,50^ annotates the intergenic rs1826907 as modifying regulatory sequences within an enhancer region associated with adipose nuclei (Fig. 2, Supplementary Table 8). rs1789891 and rs1826907 are associated with the downregulation of different *ADH* genes (*ADH1B* and *ADH1A/1C*, respectively) in a variety of tissues (Table 1, Fig. 2), but are both associated with downregulatory interactions in subcutaneous adipose tissue. This may be explained by the fact that rs1789891 and rs1826907 alter regulatory binding motifs for many transcription factors. Notably, rs1789891 is predicted to decrease binding affinity for Ap-1, Pbx-3, Nf-Y, Rfx5 and Sp2 transcription factors, whereas rs1826907 is predicted to increase binding affinity for the same transcription factors (Supplementary Table 9). All of these transcription factors are transcribed in both subcutaneous and visceral adipose tissue (GTEx version 8^17^). Similarly, rs1789891 is predicted to increase binding affinity for Sox at one known motif, whereas rs1826907 is predicted to increase binding affinity at nine alternative Sox motifs (Supplementary Table 9). This provides a potential mechanistic explanation for the observed but coordinated differences in eQTL effects.

Rs2066702 is an exonic variant within *ADH1B* causes a missense mutation^51^ and is an eQTL associated with downregulation of *ADH1A* in subcutaneous fat. This is the only interaction for this eQTL in any gene or tissue (Supplementary Table 4, Supplementary Fig. 5). Notably, the locus marked by rs2066702 has both enhancer (H3K4me1, H3K27ac) and promoter (H3K9ac) histone marks in adipose nuclei (Supplementary Tables 7 and 8). However, rs2066702 only alters one regulatory motif, GATA, and the predicted binding affinity was not substantially changed (Supplementary Table 9).

rs1789882, rs1693457 and rs904092 are in high linkage disequilibrium (LD) and are associated with upregulation of *ADH1A* transcription in subcutaneous adipose (Fig. 2, Supplementary Fig. 4). rs1693457 alters a Sox binding motif, predicted to cause increased binding affinity with the transcription factor (Supplementary Table 9). rs1789882 has alterations in Bdp1 and Stat4 motifs that are predicted to substantially increase the binding affinity to these transcription factors (Supplementary Table 9). rs1693457 and rs1789882 are also associated with both enhancer (H3K4me1, H3K27ac) and promoter (H3K4me3, H3K9ac) adipose-specific histone marks (Supplementary Tables 7 and 8). These findings are suggestive of a significant regulatory region located within the *ADH1B* gene.

rs12639833, rs2173201, rs1614972 and rs2241894 are all significantly associated with the upregulation of *ADH1C* in subcutaneous adipose tissue. The four SNPs have a similar pattern of associated interactions with *ADH1C* across tissues, with rs12639833, rs2173201 and rs2241894 having interactions with the same 8 tissues, and rs1614972 having associated interactions with fewer (n = 3). All four of these SNPs are in high LD (Supplementary Fig. 5), which likely explains the similarity in the regulatory interactions we found. The exonic variant rs2241894 has the only annotated enhancer histone mark (H3K27ac) for these SNPs in adipose nuclei (Supplementary Tables 7 and 8) but does not alter any regulatory motifs, unlike the other three (Fig. 2, Supplementary Table 9).

When looking across all gene interactions, only 7 eQTLs were identified to regulate gene expression in visceral adipose, compared to 23 in subcutaneous adipose tissue. By contrast, more than 55% (13 out of 23) of the identified eQTL interactions within the subcutaneous adipose occur within the *ADH* region. Rs1789924 is the only eQTL that is associated with genetic regulation in visceral adipose regulation and downregulates *ADH1C* in both visceral and subcutaneous adipose (as well as other tissues; Table 1). The variant is annotated with enhancer (H3K4me1, H3K27ac) and promoter (H3K4me3, H3K9ac) histone marks in adipose nuclei (Supplementary Tables 7 and 8).

Collectively, these results suggest that changes to the subcutaneous adipose tissue specific regulation of the ADH locus might be a predisposing factor for AD.

### *ADH* regulation in the Gastrointestinal system

The gastrointestinal (GI) tract plays a pivotal role in the absorption of alcohol after consumption, primarily in the stomach and small intestine (20% and 80% of absorption, respectively)^14^. These tissues also have a role in primary metabolism of the ethanol through ADH isozymes, of which *ADH1B, ADH1C* and *ADH5* are highly transcribed in stomach and small intestine (GTEx version 8^17^).

68% (49 out of 72) of eQTLS associated with regulation of *ADH* gene expression were found to have regulatory effects in tissues of the GI tract (8 sigmoid colon, 4 transverse colon, 2 gastroesophageal junction, 15 esophagus mucosa, 13 esophagus muscularis, 1 minor salivary gland, 4 small intestine-terminal ileum, 2 stomach; Fig. 2, Supplementary Fig. 5). *ADH1A* expression was modified by AD associated SNPs in 5 tissues across the GI tract; *ADH1C* in 3 and *ADH4* in 1. Notably, *ADH4* expression was regulated by eQTLs that also affect *ADH1C* expression in esophagus tissue (*ADH4* in muscularis, *ADH1C* in mucosa; Fig. 2). 3 eQTLs (rs2173201, rs2241894, rs12639833) affected *ADH4* expression in both esophagus muscularis tissue and skeletal muscle, suggestive of them acting as a co-ordinated set in muscular tissues. Of these variants, rs2173201 is the only one with H3K27ac (enhancer) histone marks in esophageal tissue (Fig. 2, Supplementary Table 7 and 8) and is associated with a predicted increase in binding affinity of Dmrt2 and Irf transcription factors to the regulatory motif (Supplementary Table 9).

Similar to our observations in adipose tissue, rs904092, rs1789882 and rs1693457 show a related pattern of regulatory interactions for upregulation of *ADH1A* within GI tissues (i.e. esophagus muscularis, small intestine and sigmoid colon tissues). Moreover, each of these variants is associated with both enhancer (H3K4me1 and/or H3K27ac) and promoter (H3K4me3 and/or H3K9ac) histone marks in at least one GI tissue (Fig. 2, Supplementary Table 7 and 8). rs1693457 is an intronic SNP within *ADH1B* that is associated with changes in *ADH1A* expression and has the most tissue interactions of any eQTL we identified. Compared to rs904092, rs1693457 has additional upregulatory associations with *ADH1A* in the gastroesophageal junction and transverse colon. This is the only interaction we identified in the gastroesophageal junction. As stated earlier, the similarity between rs904092, rs1789882 and rs1693457 is likely to reflect the high LD between the SNPs and the existence of a composite regulatory element. Notably, rs1789882 is an exonic SNP within *ADH1B* that is linked to differential expression of *ADH1A*. The rs1789882 variant alters Ehf and Elf3 regulatory motifs (Fig. 2, Supplementary Table 9), transcription factor paralogs which are reported to have epithelial-specific expression in the intestine^52^. However, the change to the minor allele does not appear to substantially change the predicted binding affinity of these proteins. Rather, alterations to Bdp1 and Stat4 binding sites are predicted to increase binding affinity and these transcription factors are transcribed in all studied tissues in the GI tract (GTEx version 8^17^). rs2173201 and rs1789891 are intergenic SNPs that are located <2kbp apart but have opposing regulatory effects on *ADH* gene expression in esophagus tissues. rs2173201 is associated with *ADH1C* upregulation in esophagus mucosa; *ADH4* upregulation in *ADH4*; H3K27ac (enhancer) histone marks in esophagus cells; and is predicted to alter binding motifs as previously mentioned (Fig. 2). Conversely, rs1789891 is associated with down regulation of *ADH1C* in esophagus mucosa and lacks associated histone marks in esophagus cells (Fig. 2, Supplementary Table 8). However, rs1789891 substantially decreases binding affinity for many transcription factors including Ap-1, Irf, Nf-Y, Pbx-3, Rfx5, Sp2 (Supplementary Table 9). All of these are transcribed in a variety of GI tract tissues, as well as many other tissues in the body (GTEx version 8^17^).

rs1614972 is an example of an intronic SNP within *ADH1C*, which is associated with upregulation of *ADH1C* expression. rs1614972 only has association in a single tissue for the GI system, esophagus mucosa, and has no histone marks for this tissue. The SNP is however associated with a predicted increase in Nkx2 transcription factor binding affinity and decrease in Gr (aka Nr3c1) and Spz1 transcription factor binding affinity (Supplementary Table 9). Nkx2 and Spz1 are usually lowly transcribed in the GI tissues, whereas Nr3c1 is highly transcribed (GTEx version 8^17^).

### *ADH* regulation is altered in the cardiovascular system and lungs

**Figure 3:**
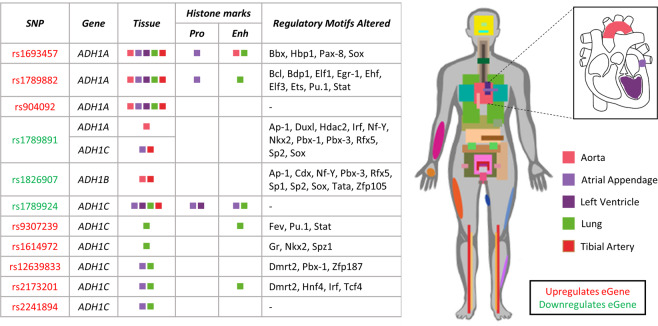
eQTL-tissue regulatory interactions associated with tissues in the cardiovascular system and the *ADH* genes. SNPs affect regulatory motifs and are present in areas of the genome that have tissue-specific histone marks that infer some regulatory function. Coloured boxes represent a significant eQTL-eGene interaction was found in the tissue and any tissue-specific histone marks, in respective columns. Tissues are represented by the same colour in the table and body diagrams. Enh = enhancer histone marks; Pro = promoter histone marks. H3K4ac and H3K27ac are grouped as enhancer histone marks and H3K4me3 and H3K9ac as promoter histone marks. Red text denotes the SNP is associated with upregulation of the eGene, whereas green text denotes associated downregulation. Histone marks and regulatory motif alterations were obtained from HaploReg (version 4.1)^49,50^.

Alcoholism is known to have links with cardiovascular disease^3^. To investigate if a genetic predisposition to change *ADH* regulation contributes mechanistically to the risk of alcohol linked cardiovascular disease, we investigated eQTLs with interactions in cardiovascular tissues (aorta, atrial appendage, left ventricle, tibial artery) and the lung, integrating HaploReg (version 4.1)^49,50^ data on regulatory motifs and histone marks (Fig. 3). Consistent with our earlier findings, we observed specific eQTLs that affected expression of *ADH1A*, *ADH1B* and *ADH1C*. Again, these variants modify known transcription factor binding sites and were associated with epigenetic modifications that indicate the presence of enhancer elements (Fig. 3, Supplementary Tables 8 and 9).

## Discussion

In this study, we integrated information on AD-associated GWAS SNPs with data on 3D genome organization, eQTLs and regulatory annotations to identify the tissue-specific regulatory roles of these variants, and thus how they potentially contribute to AD. Our findings expand our understanding of AD-associated variants, highlighting the regulatory impacts of these variants on gene expression in tissues not conventionally associated with AD.

We have shown that these variants seem to impact *ADH* expression in tissues not generally considered important for first pass metabolism of ethanol, including the lower GI tract and subcutaneous adipose tissue. Whilst these variants were not found to directly impact pathways involved in behavioural aspects of AD (i.e. in brain tissue), they suggest a potential increase in the ability of individuals to tolerate alcohol consumption through metabolic changes, therefore making them more susceptible to developing AD. This has yet to be proven clinically.

Alcohol usage is associated with many cardiovascular disorders^3^. Consistent with this, we identified associated *ADH* regulatory interactions in arteries, heart and lung tissues. This suggests that these disorders may not just be a side product of alcohol consumption, but rather they may be multimorbid due to tissue specific impacts of a sub-set of the AD associated genetic variants. This multimorbidity may extend to the obesity that is often associated with AD. Future analyses that compare the impacts of obesity/BMI associated variants with those of AD will help to resolve whether there are shared regulatory mechanisms between AD and obesity.

We identified just one association in liver tissue, a trans-acting upregulatory interaction between rs4699741 (on chromosome 4, just beyond the *ADH* locus) and *SCARB2*. *SCARB2* encodes LIMP-2 which functions as part of lysosome and endosome assembly and organisation^53^ and has not previously been linked to AD. Notably, we observed upregulation of *CYP4B1* was associated with rs2094081 in the stomach. *CYP2E1* has previously been linked to AD^54^ and is known to be activated in high concentrations of blood alcohol^14,18^. *CYP4B1*, is also a member of the cytochrome p450 family of proteins (known to play a role in response and toxicity to many drugs) and functions as part of arachidonic or fatty acid metabolism^55^.

It is well known that alleles in coding regions of *ADH* genes are associated with AD^10,14,22^. Alcoholism risk is thought to involve elevation of acetaldehyde either through increased metabolism of ethanol, or slower conversion by *ALDH*^15^. Our work indicates that non-coding regions within the ADH locus play a role in coordinating increased regulation of *ADH* genes, associated with increased AD risk. Our work also shows that upregulation of *ADH* genes can occur through a set of variants, not just a single variant, causing a co-ordinated response across multiple genes and tissues. Our results suggest that 3D chromatin structure, epigenetic regulatory histone marks and altered transcription factor binding motifs could all be playing a role in regulation of *ADH* genes. We observed upregulatory interactions with associated histone marks consistent with enhancer regions, and downregulatory interactions with associated predictions of decreased binding affinity of transcription factors due to motif alterations. Collectively these results are consistent with the existence of composite regulatory elements.

The regulatory interactions we have identified associated with SNPs in AD individuals remain putative until functionally confirmed including whether the alternative or reference alleles at the SNPs are the cause. Future GWAS studies that complete the identification of the AD associated SNPs and expanded eQTL data sets (which include analysis of immediate ancestry and gender) will enable greater refinement of the predictions we have made. Expanding Hi-C and protein expression sets will also strengthen our findings.

Some SNPs that are strongly associated by GWAS had no regulatory impacts in our assay. Our pipeline works on the assumption that the eQTLs rely on physical contact between the variant and gene it controls. However, there are other methods through which a genetic variant can impact on gene regulation that do not require this assumption be fulfilled. For example, variants in intronic regions may affect splicing activity^56^. Our approach will not identify these alternate regulatory interactions. Moreover, studying other genetic variation, including the incorporation of other genetic variants (e.g. indels, CNVs), would also contribute to strengthening our findings.

We identified eQTLs for 34 not all 73 of the AD associated SNPs. The CoDeS3D pipeline^40^ we used in this study only identifies eQTLs for a genetic variant if there are physical interactions between the variant and the gene for which it is an eQTL. However, eQTLs can impact on the expression of the gene through mechanisms that do not require a direct physical connection between the genetic variant and the gene. For example, some variants may affect splicing activity (so called splicing or sQTL variants^56^), or trans-acting factors (*e.g*. long non-coding RNAs). Therefore, the lack of a finding in this study does not mean that the variant is not important, rather it is consistent with these variants impacting on AD through a different mechanism of action.

In conclusion we have identified the potential functional impacts of specific AD-associated variants on ADH regulation and show that sets of variants can produce co-ordinated affects across ADH genes and body tissues. Knowing the positions of loci that are associated with AD does not make them clinically useful. Rather, clinical utility requires an understanding of the functional or biological impact of the genetic change. Our results emphasise the insight to be gained from detailed study of eQTL, epigenetic histone mark and motif annotation in a functional and spatial context to advance the interpretation of GWAS SNPs. These results are consistent with the growing evidence that genetic variants exert phenotypic effects through tissue-specific gene enhancers and promoters^38,57,58^. Future clinical use of this information could occur through a) development of novel tissue-specific therapeutic strategies that impact the biological and not behavioural aspects of AD; and b) stratification of patient cohorts in clinical trials. Furthermore, the integration of these data into clinical studies of AD will contribute to individualized mechanistic understandings of AD prognosis, disease development and progression.

## Methods

### GWAS SNPs

Single-nucleotide polymorphisms (SNPs) associated with alcohol dependence with *p* values <1 × 10^−6^ were downloaded from NHGRI-EBI GWAS Catalog^39^ (www.ebi.ac.uk/gwas/; 07/12/2018) (see Data and code availability). The wANNOVAR tool^59,60^ was used to perform functional annotation of SNPs. Genomic positions and annotations of SNPs were according to the human genome build hg19 release 75 (GRCh37) (see Data and code availability).

### CoDeS3D pipeline

The CoDeS3D^40^ pipeline was used to identify tissue-specific spatial interactions between regulatory regions (marked by SNPs associated with alcohol dependence) and their target genes. The CoDeS3D method we used here was previously outlined in^36^ (Supplementary Fig. 1). Briefly, 28 Hi-C chromatin interaction libraries were used in this study (Supplementary Table 2). To identify DNA fragments, the hg19 reference genome was digested with the same restriction enzyme as used in Hi-C library preparation (i.e. Mbol or HindIII). First, the location of SNPs within the restriction fragments was identified. Next, the algorithm identified the fragments that interact with the SNP-containing fragments in each of 28 Hi-C chromatin interaction libraries. These fragments were further overlapped with gene regions to identify only spatial SNP-gene pairs (where SNP-containing fragments spatially interact with gene-overlapping fragments). GENCODE transcript model version 19 was used as the reference for gene annotations. Then, we queried the GTEx database (https://gtexportal.org/, GTEx multi-tissue dataset v7, Supplementary Table 3) with the SNP-gene pairs, to identify cis- and trans-acting eQTL SNP interactions with eGenes (i.e. genes, whose tissue-specific expression changes are associated with eQTL SNP). Lastly, the Benjamini-Hochberg FDR control algorithm^40^ was applied to adjust the *p* values of the identified eQTL associations and output only significant tissue-specific eQTL SNP-eGene interactions (FDR < 0.05).

### LD analysis

Analysis of linkage disequilibrium (LD) was performed using LDlink 3.7^61^. The LDmatrix module was used to calculate LD statistics for AD-associated eQTL SNPs within *ADH* locus (GRCh37/hg19 genome assembly; SNP RS numbers based on dbSNP151; genotyping data from phase 3 (version 5) of the 1000 Genome Project; European population).

### GO enrichment and pathway analyses

Gene enrichment and pathway analyses were performed using g:Profiler^44^ (the g:GOSt module). Three Gene Ontology terms (i.e. biological process, molecular function and cellular component) were used to identify functional categories that are statistically overrepresented in the spatially regulated AD-associated eGene set. Kyoto Encyclopedia of Genes and Genomes (KEGG) database^62^ was used to identify the most impacted biological pathways. All known human genes were chosen as a statistical domain scope. The significance of the overrepresented GO terms and pathways were corrected by the SCS algorithm^44^ (adjusted *p* < 0.05).

### Regulatory annotation

Promoter-enhancer histone marks and affected regulatory motifs were obtained from the HaploReg (version 4.1)^49,50^ website for the SNP-eQTLs identified as associating with the *ADH* gene region. The presence of H3K4ac, H3K27ac, H3K4me3 and H3K9me3 epigenetic histone marks and DNase marks were recorded for all cell lines. The cell lines were then filtered for those that closely matched tissue types from the GTEx database and histone marks were recorded for the corresponding tissues for eQTLs identified. This was done for eQTLs that had significant interactions with tissues in the cardiovascular, gastrointestinal and adipose tissues (See Supplementary Tables 7 & 8). Altered regulatory motifs and reference and alternative allele scores were obtained from HaploReg (version 4.1)^49,50^ based on constructed position weight matrices (PWMs)^63,64^.

### URLs

NHGRI-EBI GWAS Catalog: https://www.ebi.ac.uk/gwas/

gnomAD v2.1.1: https://www.gnomad.broadinstitute.org/

wANNOVAR: http://wannovar.wglab.org/

CoDeS3D pipeline: https://github.com/Genome3d/codes3d-v1

GTEx Portal: https://gtexportal.org/home/

LDlink 3.725: https://ldlink.nci.nih.gov/

g:Profiler (version e95_eg42_p13_f6e58b9): https://biit.cs.ut.ee/gprofiler/

HaploReg v4: https://pubs.broadinstitute.org/mammals/haploreg/haploreg_v4.php/

## Data and code availability

Human genome build hg19 release 75 (GRCh37) was downloaded from ftp://ftp.ensembl.org/pub/release-75/fasta/homo_sapiens/dna/Homo_sapiens.GRCh37.75.dna.primary_assembly.fa.gz.

SNP genomic positions (CoDeS3D SNP database) were obtained from ftp://ftp.ncbi.nih.gov/snp/organisms/human_9606_b151_GRCh37p13/.

Gene annotations were downloaded from https://storage.googleapis.com/gtex_analysis_v7/reference/gencode.v19.transcripts.patched_contigs.gtf.

All Python and R scripts used for data analysis and visualisation are available at https://github.com/Genome3d/alcohol-dependence.

## Supplementary information

Supplementary information.

Supplementary information.
