# Supplementary material for: Genetic variants associated with alcohol dependence co-ordinate regulation of ADH genes in gastrointestinal and adipose tissues
Source: Sci Rep. 2020 Jun 18;10:9897. doi: 10.1038/s41598-020-66048-z (PMC7303195; doi:10.1038/s41598-020-66048-z)
Supplement: Supplementary file 2 — Supplementary information. [file 41598_2020_66048_MOESM2_ESM.pdf]

Supplementary figures for:

Genetic variants associated with alcohol dependence co-ordinate regulation of *ADH* genes in gastrointestinal and adipose tissues

Rebecca Hibberd[1,2,4‡], Evgeniia Golovina[1,3‡], Sophie Farrow[1], Justin M. O'Sullivan[1,2,3*]

[1] Liggins Institute, The University of Auckland

[2] MRC Lifecourse Epidemiology Unit, University of Southampton

[3] A Better Start National Science Challenge

[4] Natural Sciences, Faculty of Environmental and Life Sciences, University of Southampton

[‡] These authors contributed equally

*corresponding author: justin.osullivan@auckland.ac.nz.

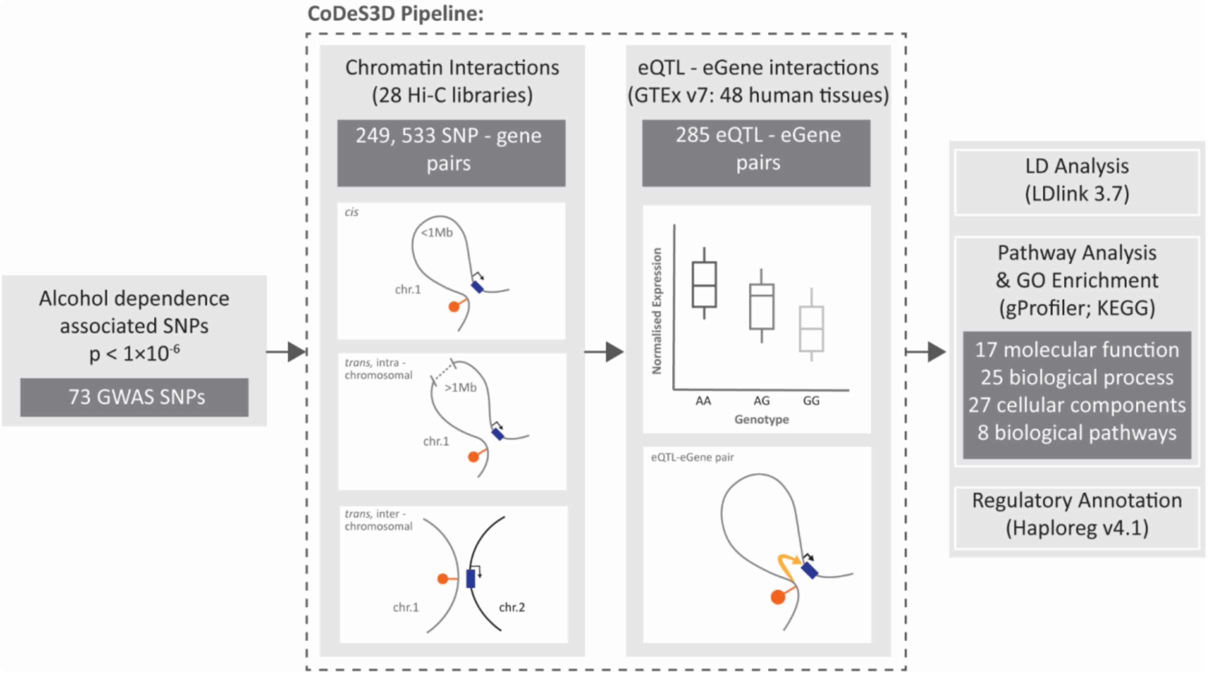

Supplementary Figure 1: Cartoon outlining the analysis performed in this study. 73 AD-associated GWAS SNPs were analysed using the CoDeS3D algorithm. First, restriction fragments containing AD-associated SNPs were identified. In total, 28 Hi-C libraries were interrogated to identify genes that spatially interact (in cis- and trans-) with the SNP-containing restriction fragments. Next, the identified spatial SNP-eGene pairs were used to query GTEx v7 (dbGaP accession phs000424.v7.p2). The Benjamini-Hochberg FDR algorithm was applied to adjust the p values of the resulting eQTL associations to identify 285 significant (FDR < 0.05) tissue-specific spatial SNP-eGene interactions. These 285 significant interactions were further processed in the downstream analyses (LD, Pathway and GO Enrichment analyses and Regulatory Annonation).

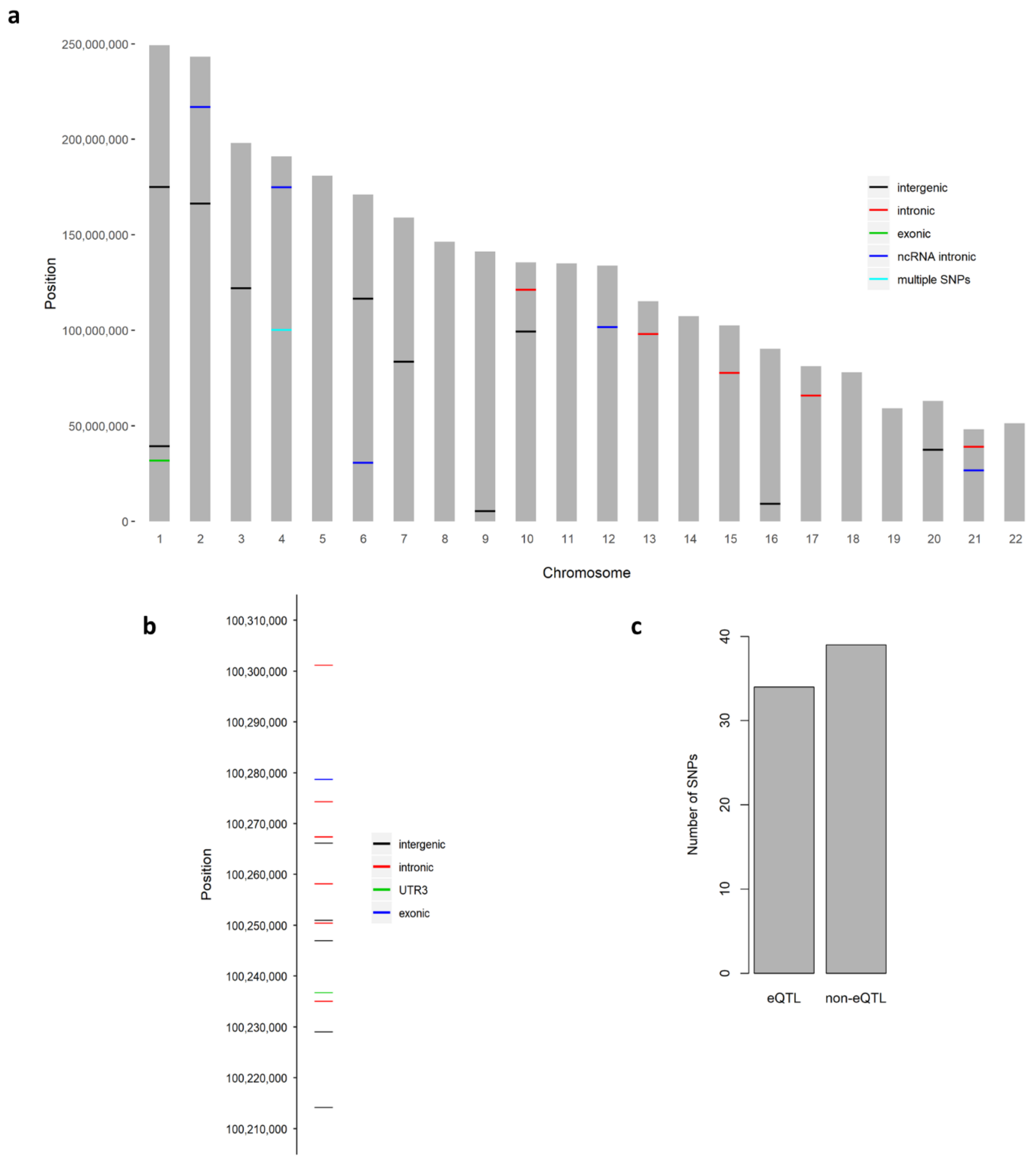

Supplementary Figure 2: a) Genomic positions of the 34 eQTLs identified (16 intronic, 9 intergenic, 3 exonic, 3 ncRNA intronic, 1 upstream, 2 UTR3; Supplementary Figure 1) on chromosomes 1 to 22 of the human genome (build hg19 release 75, GRCh37). 13 eQTLs on chromosome 4 are grouped as a single line for simplicity due to their proximity in the linear sequence. Functional annotations for the SNPs obtained from wANNOVAR[52,53] are represented as different colours. b) Expanded view of a 100kb locus on chromosome 4 (chr4:100210000-100310000) showing the genomic positions of 13 eQTLs on chromosome 4. Functional annotations for the SNPs obtained from wANNOVAR[52,53] are represented as different colours. c) Not all AD associated SNPs we analysed were identified as eQTLs. 34 out of the 73 SNPs investigated were identified as eQTLs.

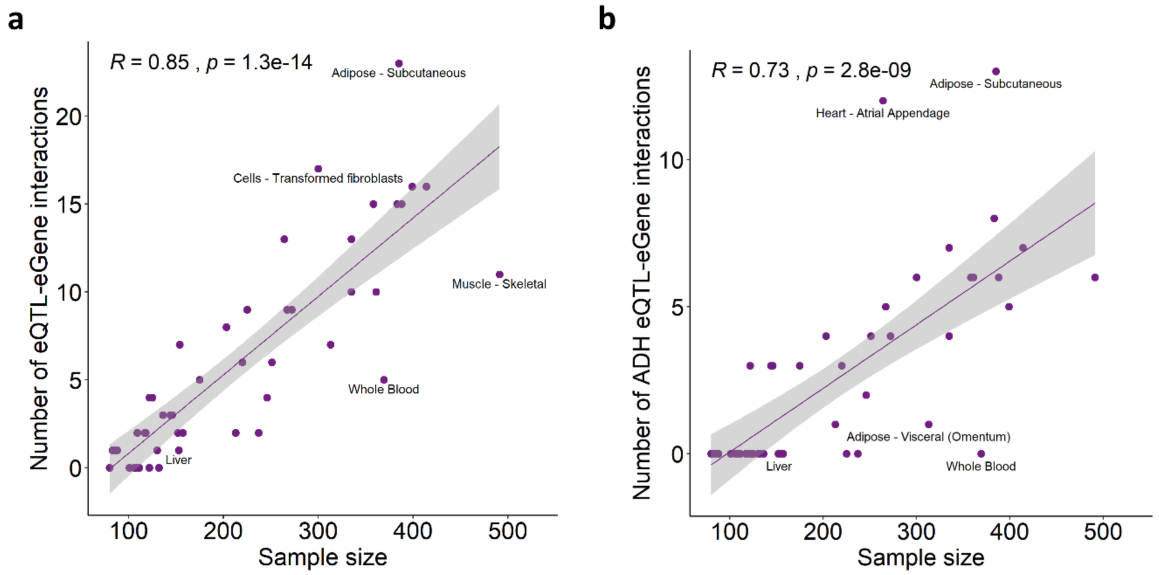

Supplementary Fig. 3: Relationships between the number of significant eQTL-eGene interactions found and the sample size for each tissue including: a) all significant eQTLs for AD; b) significant eQTLs for AD with interactions with ADH genes only. Tissues of interest are labelled.

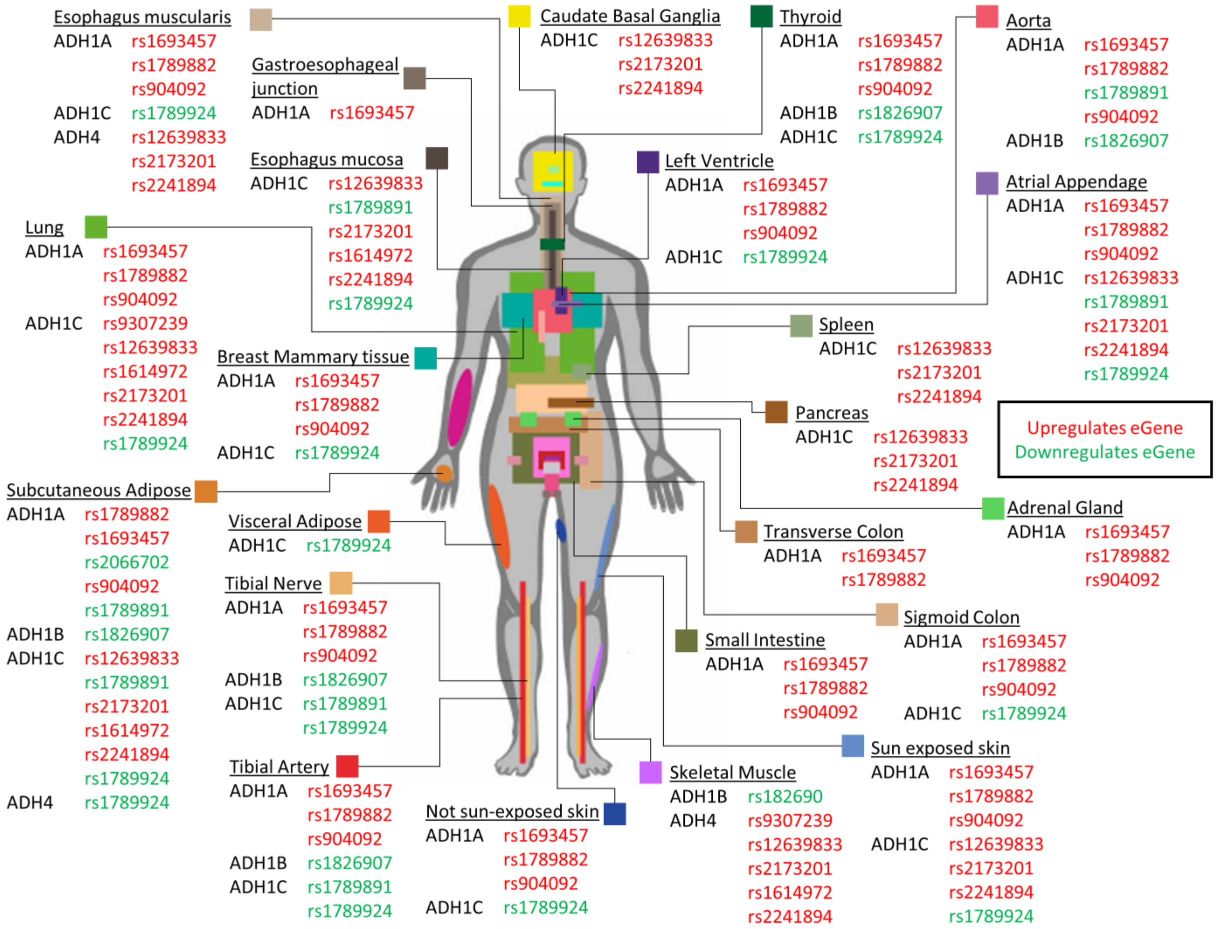

Supplementary Figure 4: Tissue-specific effects of eQTLs for ADH1A, ADH1B, ADH1C and ADH4 mapped to the human body. Red text denotes the SNP upregulates the eGene, whereas green text denotes downregulation.

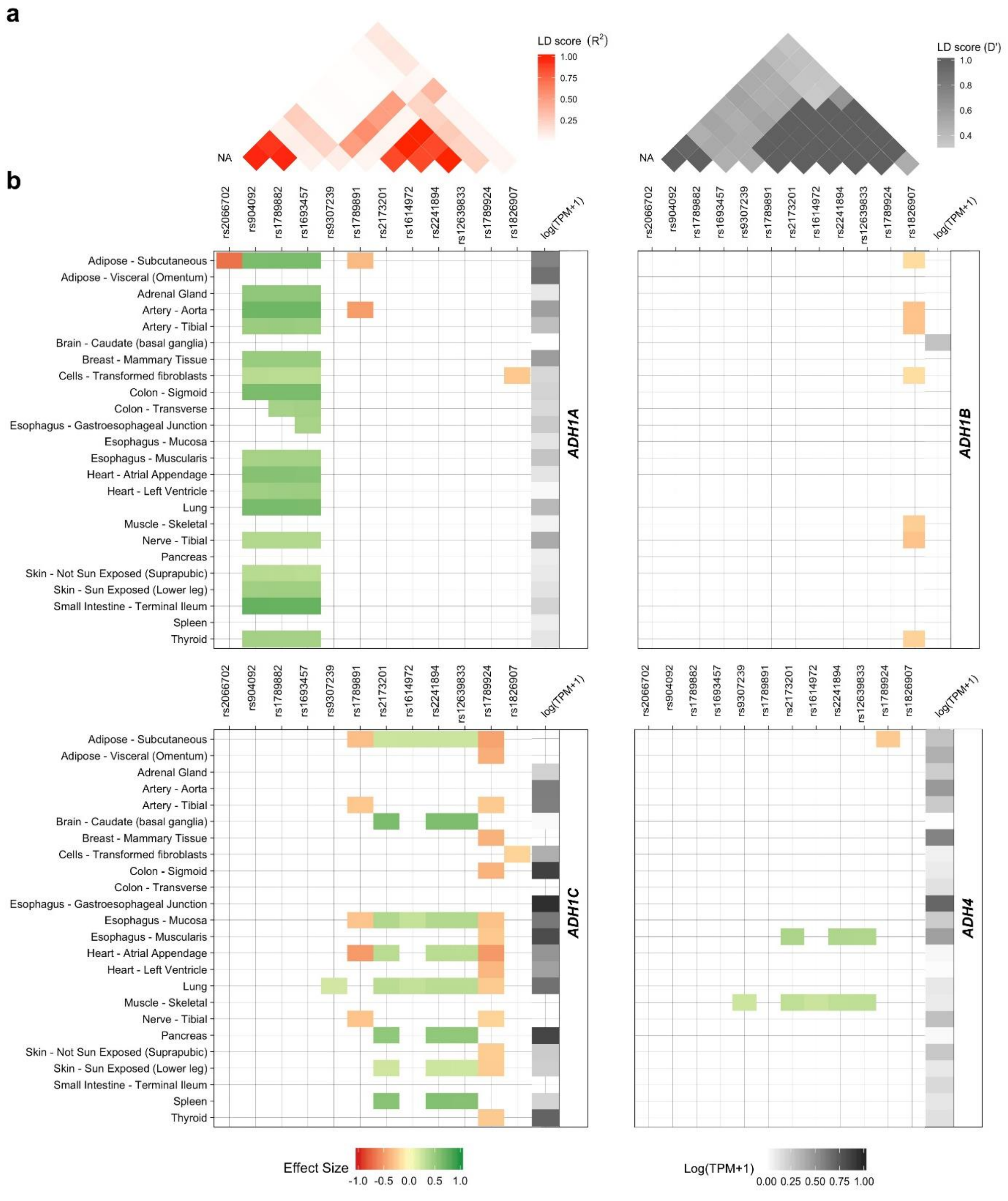

Supplementary Figure 5: eQTLs effect tissues in different patterns and have tissue-specific and LD-dependent effects on genes. a) Linkage Disequilibrium (LD) analysis for the eQTLs in the ADH locus. Left shows R-squared analysis. Right shows D-prime analysis. b**)** eQTL effect sizes and transcription levels across different tissues for four genes in the ADH region obtained from GTEx version 7.
